# SOX2 functions as a molecular rheostat to control the growth, tumorigenicity and drug responses of pancreatic ductal adenocarcinoma cells

**DOI:** 10.18632/oncotarget.8994

**Published:** 2016-04-26

**Authors:** Erin L. Wuebben, Phillip J. Wilder, Jesse L. Cox, James A. Grunkemeyer, Thomas Caffrey, Michael A. Hollingsworth, Angie Rizzino

**Affiliations:** ^1^ Eppley Institute for Research in Cancer and Allied Diseases, University of Nebraska Medical Center, Omaha, Nebraska 68198-5950, USA; ^2^ Department of Pathology and Microbiology, University of Nebraska Medical Center, Omaha, Nebraska 68198-3135, USA; ^3^ Department of Biochemistry and Molecular Biology, University of Nebraska Medical Center, Omaha, Nebraska 68198-5870, USA

**Keywords:** SOX2, pancreatic cancer, MEK inhibitors, AKT inhibitors, drug resistance

## Abstract

Pancreatic ductal adenocarcinoma (PDAC) is a highly deadly malignancy. Expression of the stem cell transcription factor SOX2 increases during progression of PDAC. Knockdown of SOX2 in PDAC cell lines decreases growth *in vitro*; whereas, stable overexpression of SOX2 in one PDAC cell line reportedly increases growth *in vitro*. Here, we reexamined the role of SOX2 in PDAC cells, because inducible SOX2 overexpression in other tumor cell types inhibits growth. In this study, four PDAC cell lines were engineered for inducible overexpression of SOX2 or inducible knockdown of SOX2. Remarkably, inducible overexpression of SOX2 in PDAC cells inhibits growth *in vitro* and reduces tumorigenicity. Additionally, inducible knockdown of SOX2 in PDAC cells reduces growth *in vitro* and *in vivo*. Thus, growth and tumorigenicity of PDAC cells is highly dependent on the expression of optimal levels of SOX2 – a hallmark of molecular rheostats. We also determined that SOX2 alters the responses of PDAC cells to drugs used in PDAC clinical trials. Increasing SOX2 reduces growth inhibition mediated by MEK and AKT inhibitors; whereas knockdown of SOX2 further reduces growth when PDAC cells are treated with these inhibitors. Thus, targeting SOX2, or its mode of action, could improve the treatment of PDAC.

## INTRODUCTION

Pancreatic ductal adenocarcinoma (PDAC) is one of the most lethal malignancies. For several decades, the 5-year survival of patients with PDAC has remained at or below 7% with a median survival of less than one year for patients with locally advanced or metastatic disease [1]. In the United States, PDAC is the fourth most common cause of cancer deaths (~40,000/year), and it is predicted to become the second leading cause of cancer deaths in the United States by 2030 [2]. The high mortality of PDAC patients is due in large measure to late diagnosis of the disease when tumor resection is not feasible and resistance of PDAC to chemotherapy designed to target aberrantly regulated signaling networks. Consequently, there is a desperate need to identify new therapeutic targets that influence drug-resistance. Thus far, a wide range of genes and signaling pathways have been shown to be aberrantly activated in PDAC. The most common mutation is in the coding region of the KRAS gene, which generates constitutively activated KRAS in >90% of all PDAC [3]. These tumors are highly dependent on upregulated AKT and RAF/MEK/ERK signaling, which are downstream of KRAS [4–7]. This led to a large number of PDAC clinical trials testing AKT inhibitors (AKTi), e.g. MK-2206, and at least five MEK inhibitors (MEKi), e.g. trametinib [8]. Disappointingly, these drugs have not produced significant responses in PDAC clinical trials, which has led to the general belief that PDAC is largely resistant to AKTi and MEKi.

Recent studies in many cancers have concluded that the stem cell transcription factor SOX2 is likely to contribute to drug resistance [9–16]. SOX2 belongs to the highly conserved HMG box family of SOX transcription factors. Early studies demonstrated that SOX2 plays essential roles during embryogenesis, and it functions as a master regulator in pluripotent embryonic stem cells [17]. Moreover, the levels of SOX2 must be carefully regulated within narrow limits in pluripotent stem cells. Not only does a reduction in SOX2 disrupt the self-renewal of embryonic stem cells [18], an increase of SOX2 as small as two-fold is sufficient to block their self-renewal and trigger their differentiation [19]. Consistent with these findings, small differences in SOX2 levels also alter the efficiency of reprogramming of somatic cells into induced pluripotent stem cells [20]. Together, these studies indicate that SOX2 functions as a molecular rheostat to control crucial activities of pluripotent stem cells [21].

SOX2 is not only essential for normal stem cell function, it has also been implicated in over 20 different cancers, including cancers of the brain, breast, ovary, lung, skin, prostate and pancreas [9–16, 22–33]. SOX2 has also been implicated in the tumor-initiating cell population (the proposed cancer stem cell population) of most of these cancers [9–16, 23–36]. With some notable exceptions [34–36], support for important roles of SOX2 in these cancers has been primarily generated from the study of tumor cell lines engineered for stable overexpression of SOX2. Stable overexpression of SOX2 in cell lines derived from many types of cancer has been reported to increase tumor cell growth *in vitro* and *in vivo* [31, 37–42]. However, different results are obtained when tumor cells are engineered for inducible overexpression of SOX2. Stable overexpression of SOX2 in the prostate tumor cell line DU145 has been reported to increase cell growth both *in vitro* and *in vivo*[37]; whereas, inducible overexpression of SOX2 (3- to 5-fold) in DU145 cells inhibits tumor cell growth [43]. Similar results have been reported for breast tumor cell lines [43].

SOX2 expression increases significantly during tumor progression rising from ~ 20% in pre-malignant PanIN3 lesions to nearly 60% of poorly differentiated PDAC [22]. Subsequent studies reported that SOX2 is expressed in many different human PDAC cell lines, with high expression in some PDAC cell lines, but little or no expression in others [23]. Importantly, this study demonstrated that SOX2 expression is closely associated with putative cancer stem cell markers previously reported to be expressed by PDAC tumor-initiating cells [23]. This study also demonstrated that knocking down SOX2 in PDAC cell lines reduced their growth *in vitro*; whereas, stable expression of SOX2 in a PDAC cell line, which does not endogenously express detectable levels of SOX2, increased their anchorage-independent growth [23]. Although this study provided support *in vitro* for a critical role of SOX2 in the stemness of PDAC, the effects of SOX2 on the tumorigenicity of PDAC tumor cells were not examined.

Here, we examined the growth responses of multiple PDAC cells lines engineered for either inducible overexpression of SOX2 or inducible knockdown of SOX2. In addition to examining how altering SOX2 expression influences PDAC cell growth *in vitro*, we examined how tumorigenicity is affected when SOX2 levels are increased and decreased. Given the association reported for SOX2 in drug-resistance in several other cancers, we also examined how changes in the levels of SOX2 influence the responses of PDAC cells to MEKi and AKTi used in clinical trials.

## RESULTS

**Figure 1:**
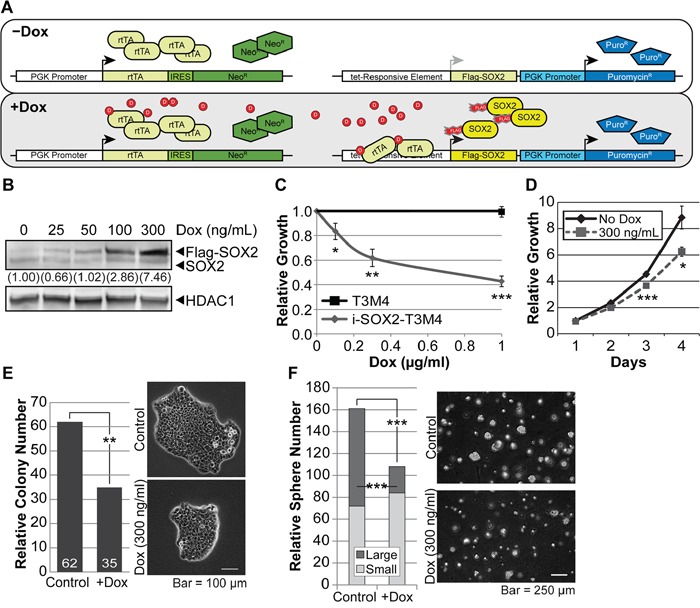
Overexpression of SOX2 in PDAC cells reduces proliferation **A.** Illustration of the two lentiviral vectors used to engineer PDAC cells for Dox-inducible, Flag-tagged SOX2 expression: one vector constitutively expresses reverse-tet transactivator (rtTA) and the second expresses Flag-SOX2, but only when its promoter is bound by rtTA complexed with Dox. **B.** Western blot analysis of SOX2 expression in whole cell extracts from i-SOX2-T3M4 cells. The overexpression of Flag-SOX2 after 24 hr of Dox treatment was compared to endogenous SOX2 in the untreated sample. HDAC1 protein was used as a loading control. **C.** Cell proliferation of i-SOX2-T3M4 was determined by MTT assay following 4 days growth at the indicated Dox concentrations. Growth in the absence of Dox was set to 1. **D.** Proliferation of i-SOX2-T3M4 cells over a 4 day period was determined by MTT assay following growth in the presence or absence of Dox (300 ng/ml). **E.** Cloning efficiency of i-SOX2-T3M4 cells was determined by the number of colonies formed after 8 days of growth in the presence or absence of Dox (300 ng/ml) as described in the Materials and Methods section. Representative photomicrographs (25X) were taken on day 8 and the cells in both panels were photographed at the same magnification. **F.** Soft agar growth in serum-free, stem cell medium and representative photomicrographs (10X) of i-SOX2-T3M4 cells in the presence or absence of Dox (300 ng/ml) after 9 days. The colonies in both panels were photographed at the same magnification. An observer unaware of sample designation scored colonies containing >8 cells in 10 random fields. Spheres larger than 50 μm were scored as “large”. Error bars represent standard deviation; statistical significance was determined by student's t-test (*p<0.05, **p <0.01, ***p<0.005). The studies shown in B, D, E, and F were repeated and similar results were obtained.

To determine how elevating the levels of SOX2 influences the behavior of PDAC cells, we engineered T3M4 PDAC cells for inducible overexpression of epitope-tagged SOX2. Epitope-tagged SOX2 enabled us to distinguish exogenously expressed SOX2 from endogenous SOX2. SOX2 was tagged at its N-terminus with a sequence that codes for a Flag-Strep tag. Previous studies have shown that placement of this tag at the N-terminus does not interfere with its function [43–47]. T3M4 cells were selected because they express SOX2 at intermediate levels, ~15-fold lower than L3.6 cells (data not shown), which have been shown previously to express SOX2 at levels significantly higher than most other PDAC cell lines [23]. Additionally, L3.6 cells express mutant KRAS (G12D);[48] whereas, T3M4 cells heterozygously express a different KRAS mutant (Q61H/WT) [49]. Using T3M4 cells, we could determine how both inducible overexpression of SOX2, as well as inducible knockdown of SOX2 (see below), influences the behavior of PDAC cells. T3M4 cells were engineered for inducible overexpression with the aid of two lentiviral vectors, which are similar to those used previously to engineer brain tumor cells for inducible expression of exogenous SOX2 [43]. One lentiviral vector codes for the expression of the reverse tet-transactivator driven by a PGK promoter, and the second lentiviral vector codes for the expression of epitope-tagged SOX2, which is driven by a doxycycline (Dox) inducible promoter (Figure 1A). After viral transduction of T3M4 cells, cells stably transduced with both lentiviral vectors were isolated as described in the Materials and Methods. These cells are referred to as i-SOX2-T3M4 cells.

### Overexpression of SOX2 reduces PDAC cell growth *in vitro* and *in vivo*

To determine how inducible elevation of SOX2 influences the *in vitro* growth of i-SOX2-T3M4 cells, we initially examined a Dox-dose response curve. As the concentration of Dox was increased, there was a dose dependent increase in the expression of Flag-SOX2. At 300 ng/ml of Dox there was ~7.5-fold increase in total SOX2 (endogenous plus exogenous SOX2) (Figure 1B). Treatment of i-SOX2-T3M4 cells with Dox over a 4 day period led to decreased cell growth at all Dox concentrations tested, reaching nearly 40% reduction in cell proliferation at 300 ng/ml of Dox (Figure 1C). A significant reduction in cell growth was evident after 72 hr (not statistically different at 48 hr, Figure 1D). As a control, we tested the effects of Dox on parental T3M4 cells. At concentrations as high as 1 μg/ml, there were no effects on the growth of parental T3M4 cells (Figure 1C). To extend these studies, we assessed the effects of elevating SOX2 on the clonal growth of i-SOX2-T3M4 cells in both monolayer culture and under anchorage-independent growth conditions. When plated at clonal densities in monolayer culture, inducible overexpression of SOX2 after 8 days significantly reduced the number of colonies, as well as the size of the colonies (Figure 1E). Importantly, even after repeated passage in the presence of Dox (> 10 passages), we failed to observe the emergence of cells that exhibited accelerated growth due to elevation of SOX2. After each passage, there was a reduction in the growth of cells treated with Dox when compared to cells cultured in the absence of Dox (data not shown). Not surprisingly, inducible elevation of SOX2 also failed to increase the growth of i-SOX2-T3M4 cells under anchorage-independent growth conditions. After treatment with Dox for 9 days in serum-free, stem cell medium, the number and size of the colonies formed in soft-agar was reduced significantly (Figure 1F). Under these conditions, there was a reduction in the total number of colonies, where the largest reduction was in the number of large colonies.

To determine whether the effects of SOX2 overexpression were PDAC cell line dependent, we engineered two additional PDAC cell lines, BxPC3 and HPAF-II, for inducible overexpression of SOX2. BxPC3 cells endogenously express SOX2 at levels ~5-fold higher than T3M4 cells; whereas, HPAF-II cells express endogenous SOX2 at levels lower than T3M4 cells (data not shown). HPAF-II cells express activated, mutant KRAS (G12D);[50] whereas, BxPC3 cells express wild-type KRAS [51, 52]. Thus, BxPC3 cells could help determine whether the effects of inducible overexpression of SOX2 were related to the KRAS status of PDAC cells. BxPC3 cells and HPAF-II cells were each transduced with the same lentiviral vector set (Figure 1A) used to engineer T3M4 cells. As shown for i-SOX2-T3M4, we observed tunable induction of exogenous SOX2 when i-SOX2-HPAF-II cells and i-SOX2-BxPC3 were exposed to increasing concentrations of Dox (Supplementary Figure 1). In addition, at all Dox concentrations tested, elevation of SOX2 in i-SOX2-HPAF-II and i-SOX2-BxPC3 cells reduced both their short-term monolayer growth and their growth at clonal density (Supplementary Figure 1). Elevating SOX2 in i-SOX2-HPAF-II, led to ~40% reduction in growth. In the case of i-SOX2-BxPC3 cells, reduction in growth was smaller, but statistically significant. Importantly, under no conditions examined did we observe an increase in proliferation when SOX2 levels were elevated in three different PDAC cell lines. Altogether our studies demonstrate that inducible overexpression of SOX2 in PDAC cells reduces their growth *in vitro*.

**Figure 2:**
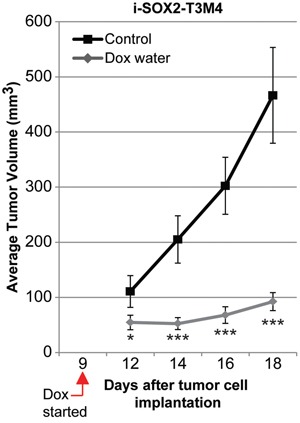
Overexpression of SOX2 in i-SOX2-T3M4 cells reduces subcutaneous tumor growth Subcutaneous tumor volumes were determined for each group of 11 mice as described in the Materials and Methods. Average tumor volumes are presented for control and Dox-treated groups. Error bars represent standard error of the mean; statistical significance was determined by student's t-test (*p<0.05, ***p<0.005).

A key property of cancer cells is tumorigenicity. To assess the impact of overexpression of SOX2 on the tumorigenicity of PDAC cells, 2.5×10^5^ i-SOX2-T3M4 cells were engrafted subcutaneously into NCr-nu/nu mice, as described in the Materials and Methods. Nine days after palpable tumors had formed, sized-matched tumors were randomly assigned to the control or the Dox-treated group. After 9 additional days, tumors in the control group (11 mice) had grown to an average > 450 mm^3^; whereas tumors in the Dox-treated group (11 mice) exhibited much less growth, reaching on average ~90 mm^3^ (~80% smaller, p<0.001) (Figure 2). In addition, tumor weight was reduced ~70% in the Dox-treated group (Supplementary Figure 2). There was also ~2-fold increase in fraction of the tumor consisting of desmoplastic stroma relative to that observed in the control tumor group, which were not treated with Dox, as determined by smooth muscle actin staining (SMA) (p<0.05, Supplementary Figure 2). Interestingly, the proliferation marker Ki-67 was ~75% lower in the tumor cell compartment of the Dox-treated tumors compared to untreated tumors (p<0.01); whereas, Ki-67 staining was ~2-fold higher in the stromal compartments of Dox-treated tumors compared to untreated tumors (p<0.05, Supplementary Figure 2). Altogether, our findings argue that inducible overexpression of SOX2 in PDAC cells does not increase cell growth, but, in fact, reduces their growth in culture as well as their tumorigenicity.

### Knockdown of SOX2 decreases PDAC cell growth *in vitro* and *in vivo*

**Figure 3:**
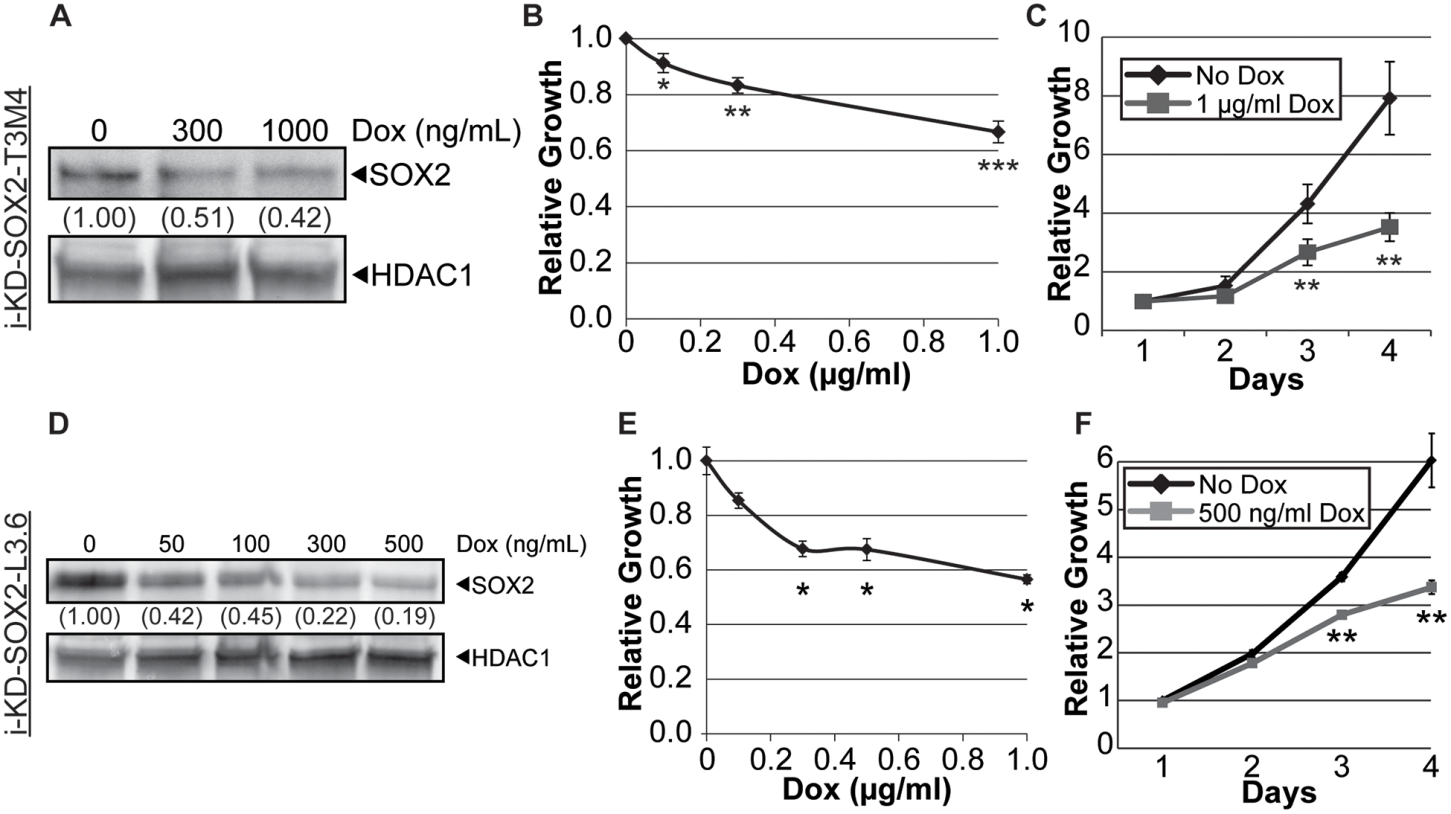
Knockdown of SOX2 in i-KdSOX2-T3M4 and i-KdSOX2-L3.6 cells reduces cellular growth **A.** Western blot analysis of SOX2 was performed using whole cell extracts from i-KdSOX2-T3M4 cells following 3 days of Dox-induction of the shRNA targeting SOX2. The level of SOX2 was compared to that in the untreated sample and HDAC1 protein was used as a loading control. **B.** Cell growth of i-KdSOX2-T3M4 was examined by MTT assay following 4 days growth at the indicated Dox doses. Growth in the absence of Dox was set to 1. **C.** Proliferation of i-KdSOX2-T3M4 cells in the presence or absence of Dox (1 μg/ml) over a 4 day period was determined by MTT assays. These studies were repeated and similar results were obtained. **D.** Western blot analysis of SOX2 was performed using whole cell extracts from i-KdSOX2-L3.6 cells following 2 days of Dox-induction of the shRNA targeting SOX2. The knockdown of SOX2 was compared to SOX2 levels in the untreated sample. HDAC1 protein was used as a loading control. **E.** Proliferation of i-KdSOX2-L3.6 was examined by MTT assay following 4 days growth at the indicated Dox concentrations. **F.** Growth of i-KdSOX2-L3.6 cells over 4 days was determined by MTT assay following growth in the presence or absence of Dox (500 ng/ml). Error bars represent standard deviation and p values were determined by student's t-test (*p<0.05, **p<0.01, and ***p<0.005). The studies in shown in A, C, D, and F were repeated and similar results were obtained.

We also assessed the impact of knocking down SOX2 on the growth and tumorigenicity of T3M4 cells. For this purpose, T3M4 cells were transduced with a single lentiviral vector that codes for an inducible promoter driving expression of a SOX2 shRNA, as well as coding for constitutive expression of the reverse tet-transactivator that is capable of binding to the Dox-inducible promoter when Dox is added to the culture medium. Productively transduced T3M4 cells, referred to as i-KdSOX2-T3M4 cells, were isolated as described in the Materials and Methods. Treatment of these cells with increasing concentrations of Dox led to dose dependent reductions in the expression of endogenous SOX2 protein (Figure 3A) and dose dependent reductions in cell growth (Figure 3B). After 3 days of growth, there was a statistically significant reduction of growth, reaching >50% inhibition after 4 days (Figure 3C) when SOX2 was reduced ~60% (Figure 3A). As discussed below, treatment with Dox at this concentration also reduced the number of colonies as well as the size of colonies when plated at clonal density in monolayer culture. Additionally, a second, independent shRNA lentiviral vector was used in T3M4 cells to validate that observed effects were due to the knockdown of SOX2. As described above, increasing the concentration of Dox resulted in dose-dependent reductions in SOX2 protein expression and in cell growth after 4 days when using this second shRNA vector; however, this shRNA was less effective at knocking down SOX2 (~40% reduction) and less effective at reducing growth (<30%, Supplementary Figure 3). Thus, in the studies described below, the cells engineered with the first SOX2 shRNA were used.

Additionally, we examined whether knocking down SOX2 in another PDAC cell line would also alter their growth. For this purpose, L3.6 cells, which express high levels of SOX2, were transduced with the same Dox-inducible SOX2 shRNA lentiviral vector used to generate i-KdSOX2-T3M4 cells. Treatment of i-KdSOX2-L3.6 cells with increasing concentrations of Dox led to a dose dependent decrease in the expression of SOX2 protein and a decrease in the growth of the cells in monolayer culture (Figure 3D–3F).

**Figure 4:**
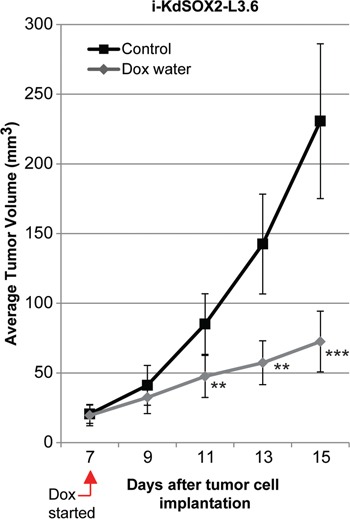
Knockdown of SOX2 in i-KdSOX2-L3.6 cells reduces subcutaneous tumor growth Subcutaneous tumor volumes were determined for each group of 7 mice as described in the Materials and Methods. Average tumor volumes are presented for control and Dox-treated groups. Error bars represent standard error of the mean; statistical significance was determined by student's t-test (**p <0.01, ***p<0.005).

Next, we assessed the impact of knocking down SOX2 on the tumorigenicity of i-KdSOX2-L3.6 cells. i-KdSOX2-L3.6 cells were engrafted subcutaneously into NCr-nu/nu mice. Once palpable tumors had formed by engrafted i-KdSOX2-L3.6 cells, mice with sized-matched tumors were randomly assigned to the control or the Dox-treated group. After an additional 8 days, the tumors in the control group increased from an average of 20 mm^3^ to an average of 230 mm^3^; whereas tumors in the Dox group increased from an average of 20 mm^3^ to an average of 70 mm^3^ – a reduction of ~70% (Figure 4). Immunohistochemical staining for the proliferation marker Ki-67 was reduced ~50% in the tumor cell compartment of the Dox-treated tumors compared to untreated tumors (p<0.05, Supplementary Figure 4). Altogether, our studies demonstrate that either increasing SOX2 (Figure 2) or decreasing SOX2 reduces the growth of tumors. Thus, the tumorigenicity of these cells is highly dependent on the expression of optimal levels of SOX2.

### Inducible modulation of SOX2 levels alters the efficacy of drugs used clinically

**Figure 5:**
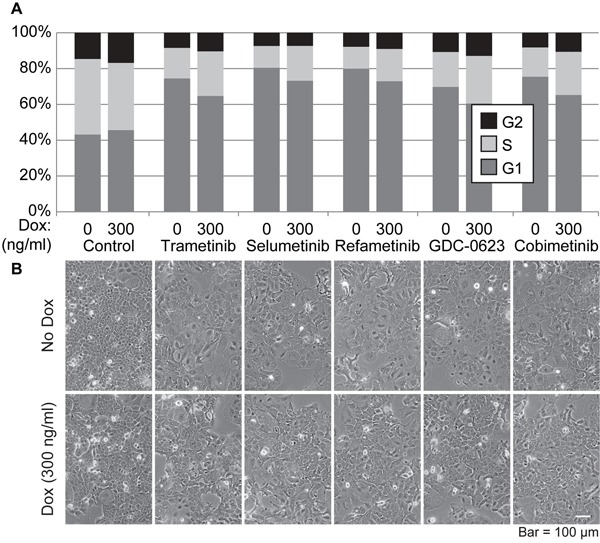
Effects of SOX2 overexpression and treatment with MEK inhibitors on the cell cycle of i-SOX2-T3M4 cells **A.** Cell cycle analysis was performed by flow cytometry on i-SOX2-T3M4 cells treated with each MEK inhibitor for 48 hr at their respective EC_50_ in the presence or absence of Dox (300 ng/ml). **B.** Representative photomicrographs of i-SOX2-T3M4 cells following growth with each inhibitor in the presence or absence of Dox (300 ng/ml). Cells in all panels were photographed at the same magnification.

Another critical property of tumor cells is their response to drugs, in particular drugs designed to target major signaling nodes activated in specific tumor types. Therefore, we tested the impact of altering the levels of SOX2 on the growth responses of PDAC cells to drugs used in PDAC clinical trials. Initially, we examined how elevating SOX2 influences the cell cycle of i-SOX2-T3M4 cells when treated with five MEKi that have been, or that are currently, used in PDAC clinical trials. For this purpose, we initially determined the EC_50_ for each MEKi exhibited by i-SOX2-T3M4 cells based on the reduction in growth over a 4 day period (Supplementary Table 1). Additionally, we confirmed the suppression of ERK1/2 phosphorylation when MEKi are used at their EC_50_ (Supplementary Figure 5). When used at their respective EC_50_, each of the MEKi led to a sizable increase in the G1 population of the cells and a sizable decrease in S-phase after 48 hr (Figure 5). As expected, elevating SOX2 by treatment with Dox also altered the cell cycle of i-SOX2-T3M4. However, there was only a modest increase in G1 and a modest decrease S-phase. Remarkably, when the cells were treated simultaneously with Dox and any of the five MEKi, we observed a partial reversal of the cell cycle changes observed with each MEKi on its own. More specifically, the increase in G1 and the reduction of S-phase observed with the MEKi was partially reduced when SOX2 levels were elevated in the cells (Figure 5A). Interestingly, each of the five MEKi induced pronounced morphological changes exemplified by significant cell spreading and this effect was also partially reversed when SOX2 was inducibly elevated (Figure 5B).

**Figure 6:**
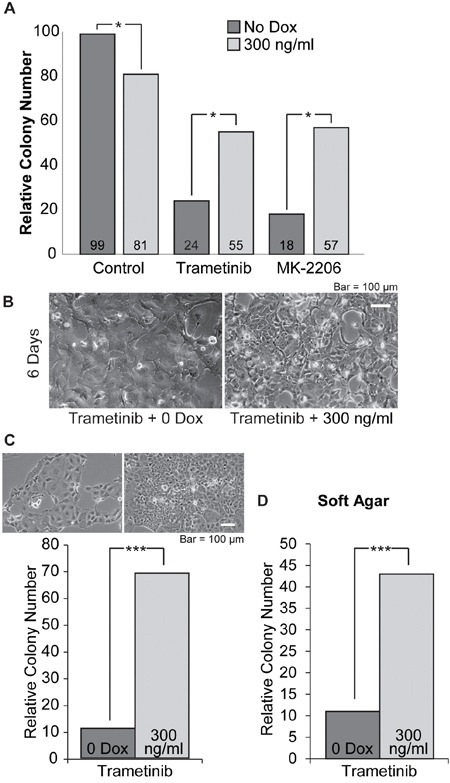
Cloning efficiency of i-SOX2-T3M4 cells is reduced by treatment with trametinib or MK-2206 and is partially reversed by overexpression of SOX2 **A.** Clonal growth was determined 8 days after plating i-SOX2-T3M4 cells at 80 cells per cm^2^. The cells were treated for 8 days with trametinib (40 nM) or MK-2206 (2 μM) in the presence or absence of Dox (300 ng/ml) where indicated. Colony number was determined by an observer unaware of sample designation in 15 random fields. These studies were repeated and similar results were obtained. **B.** i-SOX2-T3M4 cells seeded at 1.2×10^4^/cm^2^ were treated for 6 days with trametinib (40 nM) in the presence or absence of Dox (300 ng/ml) and representative photomicrographs were taken at the same magnification. **C.** After 6 days, the pre-treated cells were subcultured and plated in monolayer culture at 400 cells per cm^2^ without trametinib or Dox. After 7 days the number of colonies formed was determined as described in the Materials and Methods and representative photomicrographs were taken at the same magnification. **D.** After 6 days, the pre-treated cells were subcultured at 800 cells per cm^2^ and grown in soft agar containing serum-free, stem cell medium without trametinib and Dox. After 10 days colony numbers were determined as described in the Materials and Methods. Statistical significance was determined by student's t-test (*p<0.05 and ***p<0.005). The studies shown in A were repeated multiple times and similar results were obtained.

To more carefully assess the effects of elevating SOX2 on the growth responses of PDAC cells when treated with MEKi, we examined the clonal growth of i-SOX2-T3M4 cells cultured in the presence of one of the MEKi (trametinib) with and without Dox. For this purpose, 24 hr after the cells had been subcultured, trametinib and/or Dox were added to the cells where indicated. During the following 8 days, the cells were refed with fresh medium containing trametinib and/or Dox every other day. After 8 days of treatment, the number of colonies formed when the cells were treated with trametinib at its EC_50_ was significantly reduced. However, treatment with both trametinib and Dox led to a much smaller reduction in colony number (Figure 6A). As a control, we determined that treatment of parental T3M4 cells with Dox did not affect the dose response curves of trametinib or a second MEKi, selumetinib (data not shown).

To further evaluate the effects of trametinib on i-SOX2-T3M4 cells, the cells were cultured for 6 days at typical cell culture densities (1.2×10^4^/cm^2^) in the presence of trametinib with or without Dox. After 6 days, cells treated with trametinib or trametinib plus Dox both exhibited a change in morphology (Figure 6B) relative to untreated i-SOX2-T3M4 cells (Figure 1E), but the cells treated with trametinib on its own exhibited the most pronounced morphological change. Next, the trametinib treated and the trametinib plus Dox treated cells were subcultured and replated at clonal densities in the absence of trametinib and Dox. Although the trametinib treated cells and the trametinib plus Dox treated cells were replated at equal cell numbers, the cloning efficiency of the trametinib plus Dox treated cells was substantially higher than those treated with trametinib on its own (Figure 6C). As a control, we determined that pre-treatment with Dox on its own does not improve the cloning efficiency of i-SOX2-T3M4 cells when replated. In fact, treatment with Dox on its own for 6 days prior to replating in medium lacking Dox reduces cloning efficiency ~50% (data not shown). Interestingly, the morphology of the few colonies formed from the trametinib treated cells continued to exhibit a flattened morphology; whereas the colonies formed from the trametinib plus Dox treated cells exhibited morphology much closer to that of untreated i-SOX2-T3M4 cells. In addition, we observed a similar differential in the number of colonies formed when the trametinib, and trametinib plus Dox treated cells were replated and grown under anchorage-independent conditions in serum-free, stem cell medium (Figure 6D). Thus, even though elevating SOX2 on its own inhibits the proliferation of i-SOX2-T3M4 cells, elevating SOX2 in these cells reduces the growth inhibitory effects of trametinib under more than one condition.

To determine whether the protective effects of elevating SOX2 were cell line dependent, we examined how elevation of SOX2 influenced the clonal growth of i-SOX2-BxPC3 cells and i-SOX2-HPAF-II cells. As in the case of i-SOX2-T3M4 cells, inducible elevation of SOX2 also reduced the inhibitory effects of trametinib on the clonal growth of i-SOX2-BxPC3 cells and i-SOX2-HPAF-II cells (Supplementary Figure 6). For these studies, trametinib was used at the EC_50_ for i-SOX2-BxPC3 cells and i-SOX2-HPAF-II cells (Supplementary Table 1). Thus, the protection afforded by elevating SOX2 was not limited to i-SOX2-T3M4 cells. Equally important, the protective effect of SOX2 was not limited to trametinib. Inducible overexpression of SOX2 in i-SOX2-T3M4, i-SOX2-BxPC3, and i-SOX2-HPAF-II cells also reduced the inhibitory effects of the AKTi, MK-2206 (Figure 6A, Supplementary Figure 7). Again, MK-2206 was used at the EC_50_ for each PDAC cell line (Supplementary Table 1). Altogether, our studies show that although inducible elevation of SOX2 on its own reduces the clonal growth of three different PDAC cell lines, elevating SOX2 in these cells partially reverses the growth inhibitory effects of trametinib and MK-2206.

**Figure 7:**
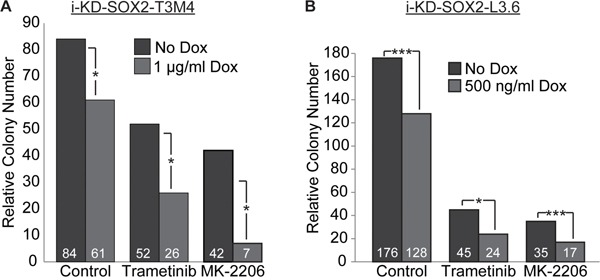
Knockdown of SOX2 in i-KdSOX2-T3M4 and i-KdSOX2-L3.6 cells reduces cloning efficiency and enhances the growth inhibiting effects of trametinib and MK-2206 **A.** Clonal growth was determined by subculturing i-KdSOX2-T3M4 cells at 80 cells per cm^2^. After 8 days of treatment with trametinib (40 nM) or MK-2206 (2 μM) in the presence or absence of Dox (1 μg/ml), colony numbers were determined. **B.** Cloning efficiency of i-KdSOX2-L3.6 cells was determined after 8 days of treatment with trametinib (20 nM) or MK-2206 (200 nM) in the presence or absence of Dox (500 ng/ml. Statistical significance was determined by student's t-test (*p<0.05 and ***p<0.005). The studies shown in A and B were repeated and similar results were obtained.

Finally, we examined whether knocking down SOX2 in PDAC cells would lead to further reduction in growth when the cells were treated with trametinib or MK-2206. Initially, we addressed this question using i-KdSOX2-T3M4 cells. As in the case of i-SOX2-T3M4 cells, treatment of i-KdSOX2-T3M4 cells with trametinib or MK-2206 each reduced the number and the sizes of the colonies that formed when the cells were plated at clonal densities (Figure 7A). Importantly, knocking down SOX2 in conjunction with trametinib or MK-2206 led to a further reduction in the number of colonies that formed. Like i-KdSOX2-T3M4 cells, growth of i-KdSOX2-L3.6 cells at clonal densities was reduced by trametinib and MK-2206 (Figure 7B). Moreover, growth of these cells was reduced even further when SOX2 was knocked down and the cells were treated with drug. Thus, these findings, in conjunction with the SOX2 overexpression studies described earlier, strongly support the conclusion that SOX2 helps protect PDAC cells from the growth inhibitory effects of MEKi and AKTi.

## DISCUSSION

Recent work has shown that SOX2 is not only expressed in ~20 different types of human cancer [9–16, 22–33], it also appears to influence drug resistance in at least six of these cancers. [9–16] SOX2 had been shown to be expressed in PDAC [22, 23], but its roles in tumor growth and drug resistance had not been examined prior to work described here. In this study, we demonstrate that either increasing or decreasing levels of SOX2 in PDAC cells reduces growth both *in vitro* and *in vivo*. Equally important, we demonstrate that elevating the levels of SOX2 reduces the efficacy of several MEKi, including trametinib, and the AKTi MK-2206, which have thus far yielded disappointing patient responses in PDAC clinical trials. Our studies indicate that the effects of SOX2 on the responses to trametinib and MK-2206 are not dependent on the mutation status of KRAS. As discussed below, targeting SOX2 or its mode of action could improve the effectiveness of these drugs against PDAC.

Prior to the work described here, stable overexpression of SOX2 in a PDAC cell line had been shown to increase cell proliferation *in vitro*. We reexamined the role of SOX2 in PDAC, because we had previously determined that inducible elevation of SOX2 in various types of tumor cells leads to growth inhibition rather than growth promotion [43]. Importantly, the work described here demonstrates that this is also true for PDAC cells. Specifically, we demonstrate that inducible elevation of SOX2 in three different PDAC cell lines *in vitro* leads to growth inhibition, rather than growth stimulation. We also determined that increases in SOX2 lead to a reduction in tumorigenicity. Under no conditions was growth observed to increase when SOX2 levels were elevated from an inducible promoter.

There may be several possible reasons why inducible overexpression leads to growth inhibition of PDAC cells, whereas stable overexpression of SOX2 can lead to increased cell proliferation. However, the most likely explanation lies in the methods used to derive the genetically engineered cells. Cells engineered for inducible overexpression were established via drug selection of virally transduced cells, which occurs at high frequency (>70%), prior to any alterations in the overexpression of SOX2. In contrast, cells engineered for stable overexpression of SOX2 undergo drug selection in the presence of elevated levels of SOX2. Consequently, cells that grow slowly in the presence of elevated SOX2, as we have shown is the case for three different PDAC cell lines, will be lost during the period of drug selection as the faster growing cell population expands. Inevitably, cells that survive drug selection in the presence of elevated SOX2 will represent a subpopulation of the parental PDAC cell population. Importantly, our studies argue that this subpopulation is likely to represent a very small minority of PDAC cells. This is especially clear in the case of i-SOX2-T3M4 cells. Continual growth of these cells in the presence of Dox for >10 passages failed to lead to the emergence of cells that grow faster due to the elevation of SOX2.

Although inducible elevation of SOX2 leads to PDAC growth inhibition *in vitro* as well as a substantial reduction in tumor growth, this does not indicate that SOX2 plays little or no role in promoting the growth of PDAC. Previous studies had shown that knockdown of SOX2 in four different PDAC cells lines reduces growth *in vitro* [23]. In the work presented in our study, we not only demonstrate that knockdown of SOX2 reduces growth *in vitro*, we also demonstrate that tumor growth of i-KdSOX2-L3.6 cells is reduced when SOX2 is knocked down *in vivo*. Thus, SOX2 is clearly required for the growth of PDAC both *in vitro* and *in vivo*. Equally important, our studies indicate that endogenous levels of SOX2 in PDAC cells are optimized for maximum growth, as both increases and decreases in SOX2 reduce PDAC cell growth. Hence, SOX2 functions as a molecular rheostat in the control of PDAC cell proliferation. Coupled with our demonstration that this is also true for ES cells [19] and four other tumor cell types [43], we suggest that this is a defining feature of SOX2.

The finding that SOX2 levels need to be maintained at optimal levels was first described in ES cells. In these cells, knockdown of SOX2 or a 2-fold increase in SOX2 disrupts the self-renewal of ES cells and triggers their differentiation [19, 45]. The need to maintain SOX2 levels within narrow limits is not surprising when one examines the SOX2-interactome in different cell types. Proteomic analysis of the SOX2-interactome in ES cells, as well as medulloblastoma cells and glioblastoma cells, indicates that SOX2 associates in high molecular weight protein complexes with a large and diverse set of nuclear proteins [46, 47, 53]. In ES cells, SOX2 is part of a highly integrated transcription circuitry that involves multiple master regulators known to control the self-renewal and pluripotency of ESC [17, 46]. Moreover, SOX2 and the other master regulators that it associates with in ES cells each form complexes with many of the same proteins. As a result, a small increase in the level of SOX2 is likely to lead to the formation of incomplete protein complexes that are essential for ES cells. Moreover, the potent biological impact of small changes in SOX2 levels seems all the more likely because SOX2 forms complexes with a wide variety of proteins involved in many critical cellular processes. In addition to transcription, SOX2 forms complexes with proteins involved in signal transduction and DNA repair [46, 47, 53]. Not surprisingly, knockdown of the RNA binding protein Musashi 2 or the deubiquitinating enzyme USP9X, which have been shown to associate with SOX2 in multiple cell types, disrupts the self-renewal of ES cells and inhibits the growth of brain tumor cells [47].

Although SOX2 levels must be maintained at optimal levels in PDAC cells, increases in the levels of SOX2 undoubtedly occur during oncogenesis. In this regard, the SOX2 gene is amplified in several cancers, [41, 54–56] and high levels of SOX2 correlate with poor prognosis in many cancers [24, 57–60]. As shown previously, SOX2 protein levels vary considerably between different PDAC cell lines [23]. In the case of T3M4 and L3.6 PDAC cells, SOX2 expression differs by ~15-fold. Thus, PDAC cells do not exhibit a single optimum for SOX2 expression. This raises a fundamental question. If SOX2 levels must be maintained within optimal limits to promote tumor growth, how can SOX2 levels rise during tumor progression? We suggest that increases in the levels of SOX2 must be accompanied by corresponding changes in other genes required for growth promotion by SOX2 and/or downregulation of genes that interfere with the action of SOX2 when its levels rise during tumor progression. Changing SOX2 levels in isolation disrupts cell function. SOX2 is by no means unique in this regard. For example, MAP3K7 and CHD1 have been shown to be co-deleted in prostate cancer and their co-deletion in ETS rearrangement-negative prostate cancers correlates with poor patient disease-free survival [61]. In a mouse xenograft model of prostate cancer, knockdown of MAK3K7 on its own had no significant effect on survival and knockdown of CHD1 on its own enhanced survival. However, combined knockdown of MAK3K7 and CHD1 led to larger tumor volumes and shorter survival [61]. Accordingly, we posit that the identification and targeting of genes that must change in concert with increases in SOX2 could provide a novel strategy for blocking, or at least, reducing the growth of tumors dependent on SOX2.

Currently, it is unknown how SOX2 reduces the effectiveness of MEKi and AKTi in PDAC cells. However, progress has been made in understanding how SOX2 is regulated in PDAC as well has how SOX2 influences the growth of PDAC cells. A recent study points to an interesting connection between SOX2 and NFATc1. Knockdown of NFATc1, which is often overexpressed in PDAC, leads to a decrease in SOX2 expression, and this appears to be due to a direct effect of NFATc1 on SOX2 transcription [62]. In other studies, stable overexpression of SOX2 in Patu8988t PDAC cells, which do not express detectable levels of endogenous SOX2, has been shown to increase expression of Twist, Snail and Slug, while decreasing the expression of E-Cadherin and ZO-1 [23]. Conversely, knocking down SOX2 in PDAC cells increases the expression of p21^Cip1^ and p27^Kip1^ [23]. Collectively, NFATc1 and SOX2 appear to work together to regulate the expression of genes involved in epithelial-mesenchymal transition and cell cycle regulation.

SOX2 not only influences tumor growth, it also influences responses of tumor cells to drugs used clinically [9–16]. In this study, we demonstrate for the first time that inducible elevation of SOX2 in three PDAC cell lines leads to a reduction in the efficacy of several MEKi, including trametinib, and the AKTi MK-2206. This is particularly interesting for two reasons. First, elevation of SOX2 on its own inhibits growth, but when SOX2 is elevated it reduces the efficacy of MEKi and AKTi. Thus, the protective effects of SOX2 against these drugs are not coupled mechanistically with the effects of SOX2 on PDAC growth. Second, knockdown of SOX2 in PDAC cells combined with drug treatment leads to further reductions in PDAC cell growth. Consequently, SOX2 appears to be a potential therapeutic target for improving the treatment of patients with SOX2-positive PDAC. Although it is generally believed that it is very difficult to develop drugs that directly interfere with the action of transcription factors, it may be practical to identify small molecule inhibitors that reduce SOX2 gene expression, block the downstream mechanisms by which SOX2 reduces efficacy of MEKi and AKTi, or, as discussed earlier, target genes that work in concert with SOX2 to promote tumor growth.

In summary, we show here that either increasing or decreasing SOX2 levels reduces the growth of PDAC cells *in vitro* and *in vivo*. Equally significant, we show that SOX2 can interfere with the ability of MEKi and AKTi to reduce the growth of PDAC cells. Going forward, it will be essential to gain a much deeper understanding of how SOX2 influences the growth of PDAC. In addition, it will be valuable to determine how SOX2 can reduce the action of MEKi and AKTi. The genetically engineered PDAC cell lines described in this study should provide a highly useful platform for addressing both of these important problems.

## MATERIALS AND METHODS

### Cell culture

T3M4, BxPC3, and HPAF-II PDAC cells have been described previously [63]. L3.6 PDAC cells were obtained from D. Billadeau (Mayo Clinic, Rochester Minn). The identity of each of these cell lines was verified by genetic analysis, which was performed by the Molecular Diagnostics Laboratory at UNMC. Stock cultures of T3M4, BxPC3, HPAF-II, and L3.6 PDAC cells and their genetically modified derivatives (see below) were cultured in Dulbecco's Modified Medium supplemented with 10% fetal bovine serum, as described previously [63]. Doxycycline (Dox, Clontech, Mountain View, CA) was suspended in phosphate buffered saline at the indicated concentrations. In all engineered lines, Flag-tagged SOX2 or SOX2 shRNA was induced by supplementing the culture medium with Dox for the times and at the concentrations indicated. Kinase inhibitors were obtained from companies listed in Supplementary Table 1. Supplementary Table 1 also contains the EC_50_ for each drug used for each cell line as determined by measurements of cell growth over a 4 day period. The cells were photographed with a Canon Rebel XTi camera at 10X and/or 25X. For cloning efficiency assays, cells were plated at clonal densities (80 cells per cm^2^) and maintained in serum containing media (as indicated above). After 8-12 days, the number of colonies (8 or more cells per colony) was determined in 15-20 random 40X fields by an observer unaware of sample designation. For replating efficiency assays, cells were grown at subconfluent densities for 6 days with or without treatment in normal media (as indicated above), at which point the cells were trypsinized and replated at clonal densities in normal media. After an additional 7 or 11 days, the number of colonies that exhibited 8 or more cells per colony was determined in 8-15 random 40X fields by an observer unaware of sample designation. Soft agar growth assays were performed in serum-free, stem cell medium, as described previously [63]. The number of spheres that exhibited 8 or more cells per sphere was determined, and spheres larger than 50 μm in diameter were scored as large. MTT assays of triplicate samples were used to assess relative cell growth, as described previously [64, 65].

### Cell engineering for SOX2 overexpression and knockdown

Cell lines were engineered for Dox-inducible SOX2 expression as described previously [43]. Separate lentiviral vectors were generated with pLVX-tetO-(fs)SOX2 and with a vector expressing the reverse tet transactivator, pLVX-Tet-On® Advanced (modified to use a PGK rather than a CMV promoter) [66]. Cells which successfully integrated these viral vectors were selected for in medium containing 5 μg/ml puromycin (P8833, Sigma-Aldrich, St. Louis, MO) for 48 hours and 300 μg/mL G418 sulfate (#631308, Clontech, Mountain View, CA) for 9-12 days, respectively. T3M4 cells were first transduced with the reverse tet transactivator lentiviral vector and a G418-resistant clone selected before infection with and selection for the pLVX-tetO-(fs)SOX2 lentiviral vector, resulting in i-SOX2-T3M4 cells. The cell lines i-SOX2-BxPC3 and i-SOX2-HPAF-II were engineered by infection with both viruses simultaneously prior to selection.

For inducible knockdown of SOX2, T3M4 and L3.6 cell lines were engineered for Dox-inducible expression of an shRNA using a TRIPZ lentiviral vector obtained from Open Biosystems (now GE Dharmacon, Lafayette, CO). This vector, RHS4696-201902991, has a mature antisense sequence of ACATGCTGATCATGTCCCG, which targets the ORF of both human and mouse SOX2. In T3M4 cells a second lentiviral vector was used independently. This second vector, RHS4696-201899634, has a mature antisense sequence of TTCTTGTCGGCATCGCGGT. The TRIPZ vector results in puromycin resistance and constitutive expression of a reverse tet transactivator as well as Dox-inducible expression of the shRNA and red fluorescent protein (RFP). I-KdSOX2-T3M4 and i-KdSOX2-L3.6 cell lines were isolated after puromycin selection, as described above. The i-KdSOX2-T3M4 cell population was further enriched by flow cytometry for cells with higher RFP expression following an 18 hour induction with 1 μg/mL Dox.

### Western blot analysis

Whole cell protein extraction buffer consists of 150 mM NaCl, 50 mM Tris-HCl (pH 7.4), 1% IGEPAL, 0.25% sodium deoxycholate, and 1 mM EDTA, which were obtained from Sigma-Aldrich. Whole cell extracts were supplemented with protease and phosphatase inhibitors for western blot analysis and protein concentrations were determined as described previously [19, 67]. Protocols for western blot analysis have been described previously [68]. SOX2 protein levels were determined with a SOX2 antibody (ab-92494, Abcam, Cambridge, MA, 1:1,000); ERK1/2 phospho-protein levels were determined with a phospho-p44/42 MAPK antibody (#9106, Cell Signaling Technology, Danvers, MA, 1:1,000). HDAC1 was used as the loading control and probed with an HDAC1 antibody (ab-7028, Abcam, 1:5,000). SOX2 and HDAC1 primary antibodies were detected with an anti-rabbit-IgG-AP secondary antibody (A3687, Sigma-Aldrich,1:10,000); Phospho-ERK1/2 primary antibody was detected with an anti-mouse-IgG-AP secondary antibody (A4312, Sigma-Aldrich,1:10,000).

### Cell cycle analysis

I-SOX2-T3M4 cells were seeded at subconfluent densities in the presence or absence of Dox (300 ng/ml) and MEKi were added the following day. After 3 days treatment with each MEKi in the presence or absence of Dox, cells were prepared for cell cycle analysis by the Telford Method, as described previously [69]. Floating cells were included in the cell cycle analysis. Flow analyses were performed by the UNMC Cell Analysis core facility.

### Determination of tumorigenicity

Female NCr-nu/nu mice (4 weeks of age) were obtained from Charles River (Wilmington, MA). All animal procedures were approved by the UNMC Institutional Animal Care and Use Committee. Where indicated, 2.5×10^5^ i-SOX2-T3M4 cells or 6.0×10^5^ i-KdSOX2-L3.6 cells were trypsinized, resuspended in 50 μl of sterile PBS, and injected subcutaneously into the flank. Tumor growth was monitored daily. After palpable tumors had formed, tumor-bearing mice were randomized to size-matched control and experimental (Dox-treated) groups. Dox-treatment for elevation or knockdown of SOX2 was accomplished by addition of Dox (2 mg/ml, Sigma-Aldrich, St. Louis, MO) to drinking water that contained 5% sucrose. Untreated mice were provided with 5% sucrose drinking water as a control. Tumor volumes were calculated based on measurements with a digital caliper at the times indicated. At the completion of the tumor growth study, mice were euthanized and tumors excised for weight measurements and immunohistochemical analysis. Exponential trend lines for tumor growth were determined using Microsoft Excel. Formalin-fixed tumor sections were paraffin-embedded and stained for hematoxylin and eosin (H&E), SMA, and Ki-67 by the University of Nebraska Medical Center Tissue Sciences Facility. H&E, SMA, and Ki-67 stained photomicrographs were captured using either an iScan Coreo Au Scanner with iScan Coreo 3.4.0 software (Ventana Medical Systems, Inc., Tucson, AZ), or a Nikon Digital Sight DS-Fi1 camera with NIS Elements 4.0. software (Nikon, Inc., Melville, NY).

Quantification of the stromal component of tumors was assessed by overlaying a grid on top of photomicrographs of SMA stained tumor tissues using Adobe Photoshop 2015.0.1. An area of 1 mm^2^ was divided into 864 squares, which were examined for positive staining, indicating stroma. Two independent tumors from each treatment condition (e.g. i-SOX2-T3M4 cells or i-KdSOX2-L3.6 cells, without Dox and with Dox) were assessed, and the percentages of SMA positive squares were averaged and standard deviation calculated for each condition. Proliferation in tumors was assessed by staining for Ki-67, and counting the number of positively stained cells out of at least 500 cells, only in the carcinoma or stromal components of each tumor growth condition. As with the stromal quantification, two independent tumors from each treatment condition were scored, and Ki-67 incidence in the stroma or carcinoma was averaged and standard deviation calculated. The student's T-test (2-tailed) was used to determine statistical significance (p < 0.05) using Microsoft Excel for Mac (15.20).

## SUPPLEMENTARY FIGURES AND TABLES


