# Supplementary material for: SOX2 functions as a molecular rheostat to control the growth, tumorigenicity and drug responses of pancreatic ductal adenocarcinoma cells
Source: Oncotarget. 2016 Apr 26;7(23):34890–906. doi: 10.18632/oncotarget.8994 (PMC5085197; doi:10.18632/oncotarget.8994)
Supplement: Supplementary file 1 [file oncotarget-07-34890-s001.pdf]

SUPPLEMENTARY FIGURES AND TABLE

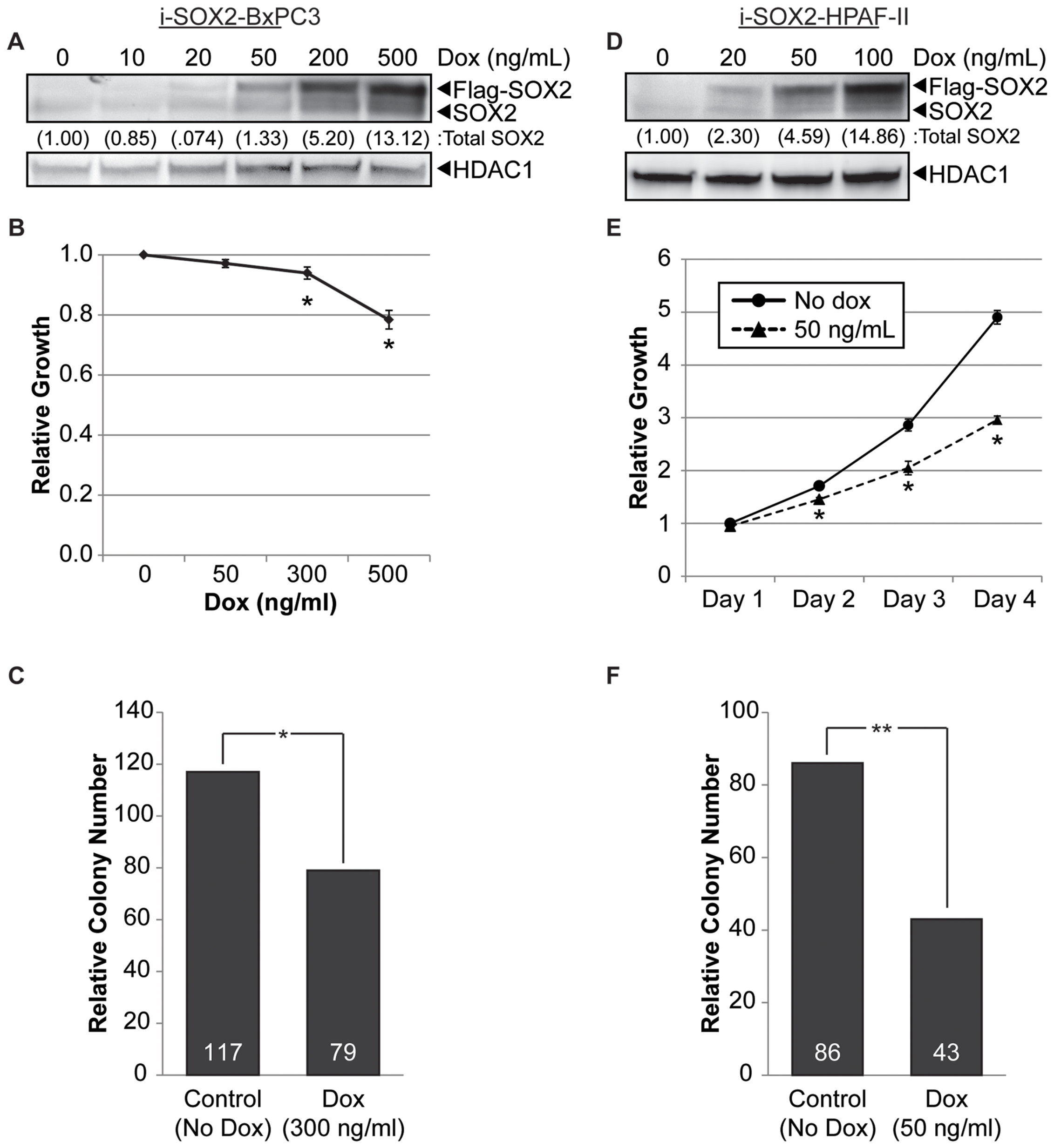

Supplementary Figure S1: Overexpression of SOX2 in PDAC cells reduces cellular proliferation. A. Western blot analysis of SOX2 expression in whole cell extracts from i-SOX2-BxPC3 cells following 3 days of Dox-induction of the transgene. The overexpression of Flag-SOX2 was compared to endogenous SOX2. HDAC1 protein was used as a loading control. **B.** Proliferation of i-SOX2-BxPC3 cells was determined by MTT assay following 4 days growth at the indicated Dox concentrations. Growth in the absence of Dox was set to 1. **C.** Clonal growth of i-SOX-BxPC3 cells was determined after 8 days in the presence or absence of Dox (300 ng/ml). **D.** Western blot analysis of SOX2 expression in whole cell extracts from i-SOX2-HPAF-II cells following 2 days of Dox-treatment. The overexpression of Flag-SOX2 was compared to endogenous SOX2. HDAC1 protein was used as a loading control. **E.** Growth of i-SOX2-HPAF-II cells over 4 days was determined by MTT assay following growth in the presence or absence of Dox (50 ng/ml). **F.** Clonal growth of i-SOX2-HPAF-II cells was determined after 8 days in the presence or absence of Dox (50 ng/ml). Error bars represent standard deviation; statistical significance was determined by student's t-test (*$p<0.05$, **$p<0.01$). The studies shown in A, C, D, and F were repeated and similar results were obtained.

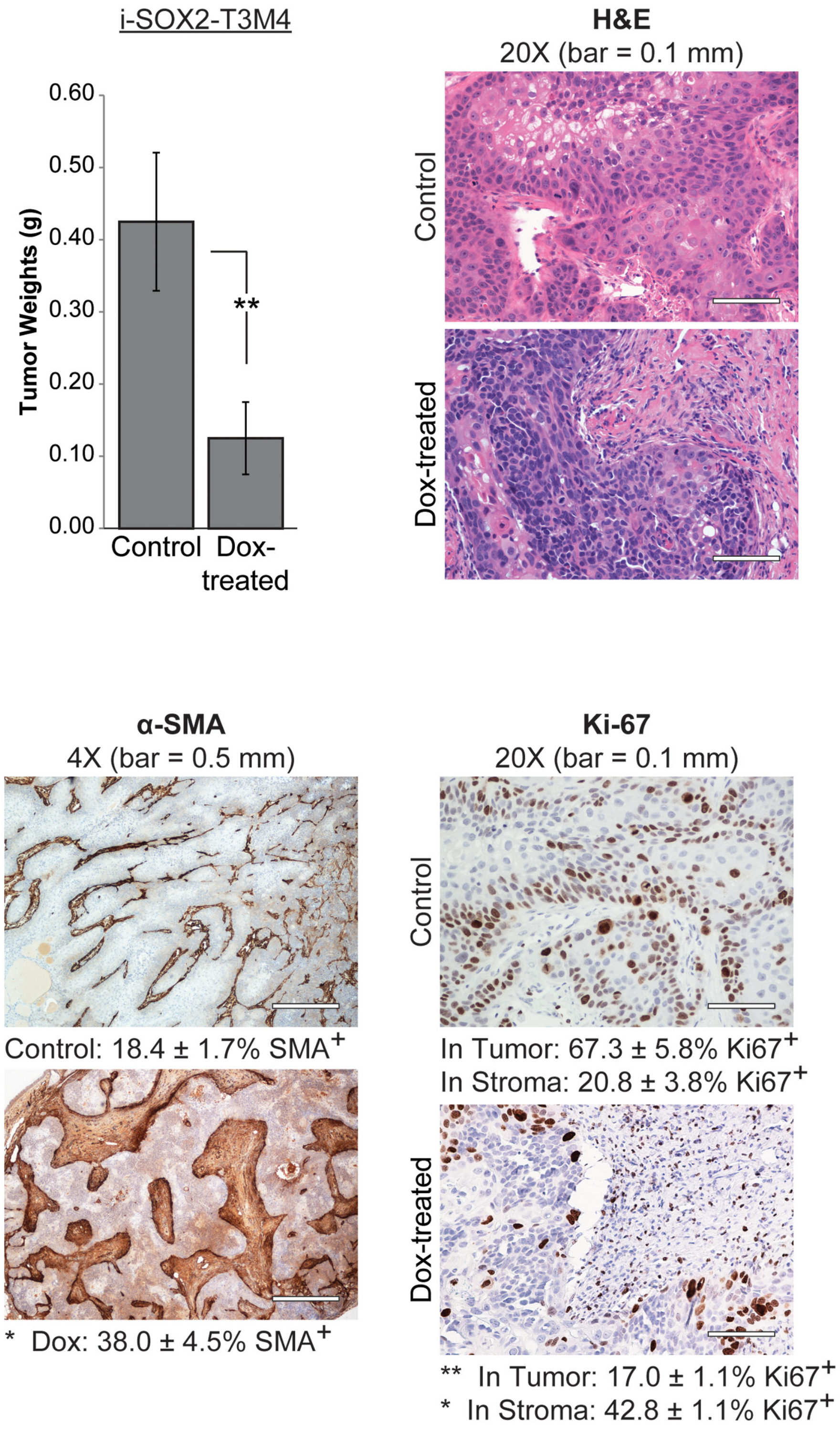

Supplementary Figure S2: Overexpression of SOX2 in i-SOX2-T3M4 cells reduces subcutaneous tumor growth. A. Tumor weights were measured following excision after a total 19 days of tumor growth. Subcutaneous tumor sections were stained and quantified for **B.** H&E, **C.** SMA, and **D.** Ki-67 as described in the Materials and Methods. Error bars represent standard deviation; statistical significance was determined by student's t-test (*p<0.05, **p <0.01).

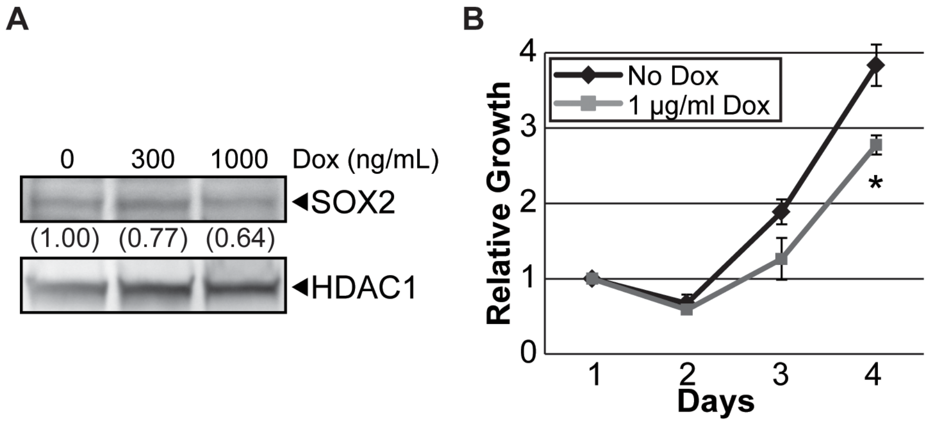

Supplementary Figure S3: Knockdown of SOX2 with a second shRNA vector cells reduces cellular growth. A. Western blot analysis of SOX2 was performed using whole cell extracts from T3M4 cells engineered with a second shRNA targeting SOX2 following 2 days of Dox-induction. The level of SOX2 was compared to that in the untreated sample and HDAC1 protein was used as a loading control. **B.** Proliferation of T3M4 cells engineered with the second SOX2 shRNA in the presence or absence of Dox (1 µg/ml) over a 4 day period was determined by MTT assays. Error bars represent standard deviation and p values were determined by student's t-test (*p<0.05).

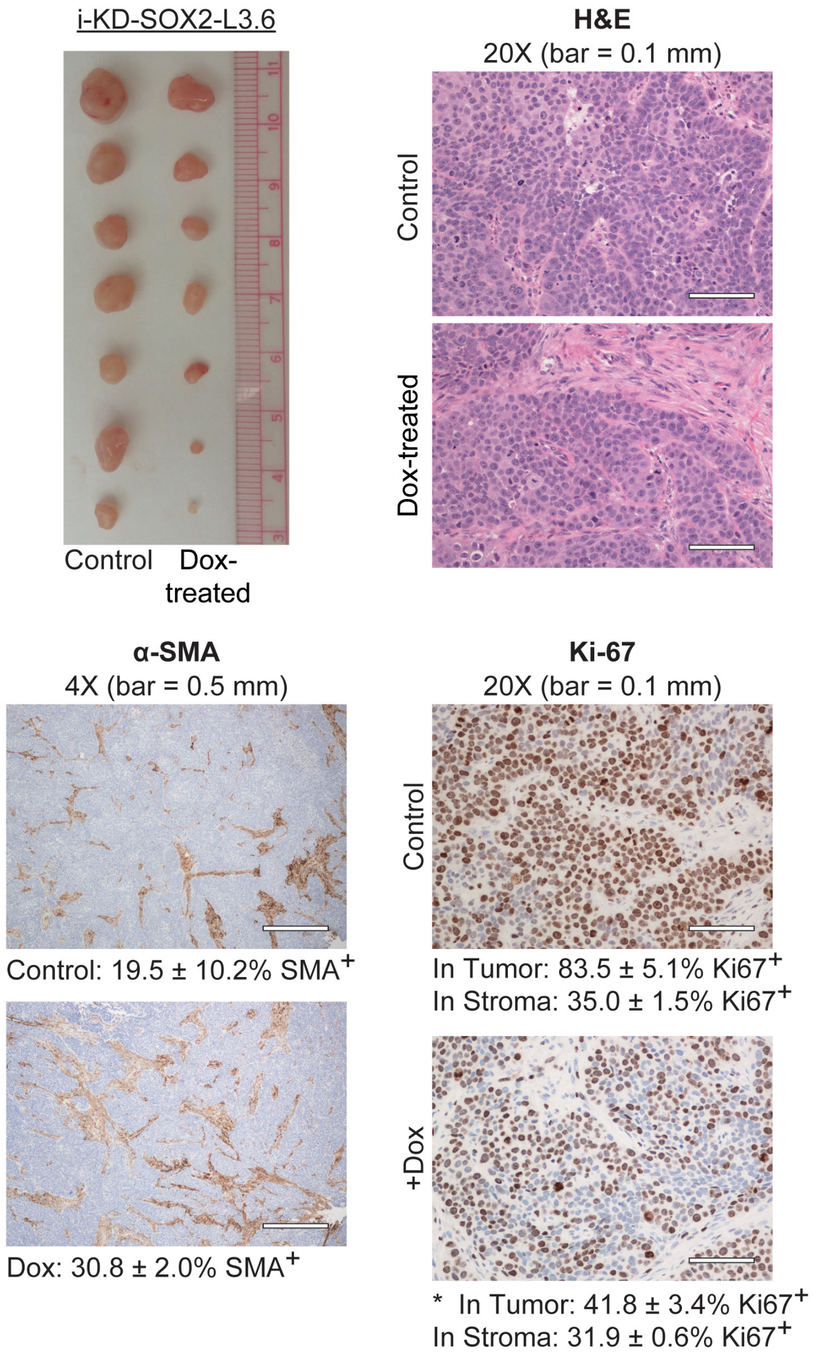

Supplementary Figure S4: Knockdown of SOX2 in i-KD-SOX2-L3.6 cells reduces subcutaneous tumor growth. A. Tumor were excised after a total 15 days of tumor growth. Subcutaneous tumor sections were stained for **B.** H&E, **C.** SMA, and **D.** Ki-67 as described in the Materials and Methods. Staining was quantified and statistical significance was determined by student's t-test (*p<0.05, **p <0.01).

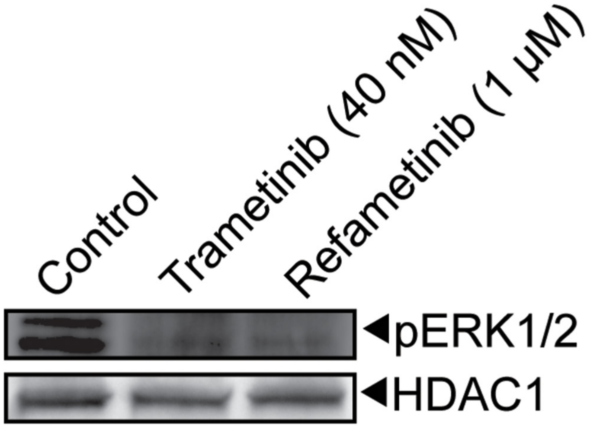

Supplementary Figure S5: Phosphorylation of ERK is suppressed following MEK inhibition. Western blot analysis of ERK phosphorylation in whole cell extracts from i-SOX2-T3M4 cells treated with the MEK inhibitors trametinib and refametinib for 48 hours at their EC_{50}. HDAC1 protein was used as a loading control.

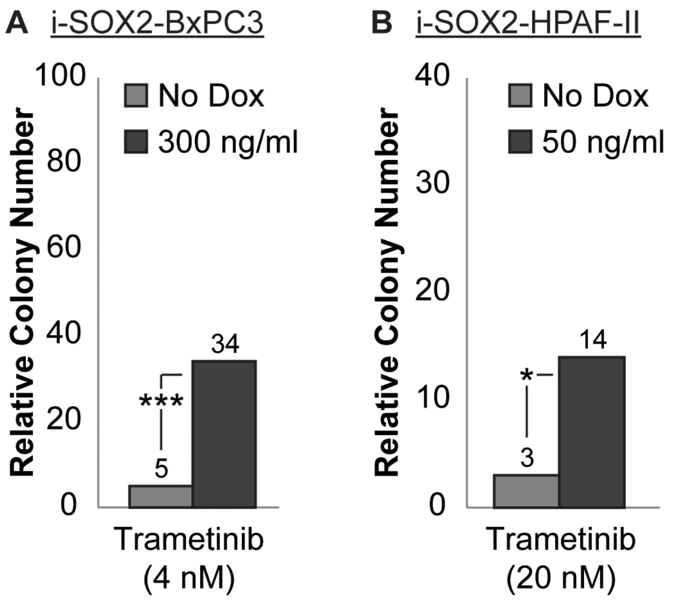

Supplementary Figure S6: Cloning efficiency of PDAC cells is reduced by treatment with trametinib and partially reversed by overexpression of SOX2. I-SOX2-BxPC3 **A.** and i-SOX2-HPAF-II **B.** cells were subcultured at 80 cells per cm^2. After 8 days treatment with trametinib (4 nM and 20 nM, respectively) in the presence or absence of Dox (300 ng/ml and 50 ng/ml, respectively) colony numbers were determined. Statistical significance was determined by student's t-test (*$p<0.05$, ***$p<0.005$). The studies shown in A and B were repeated and similar results were obtained.

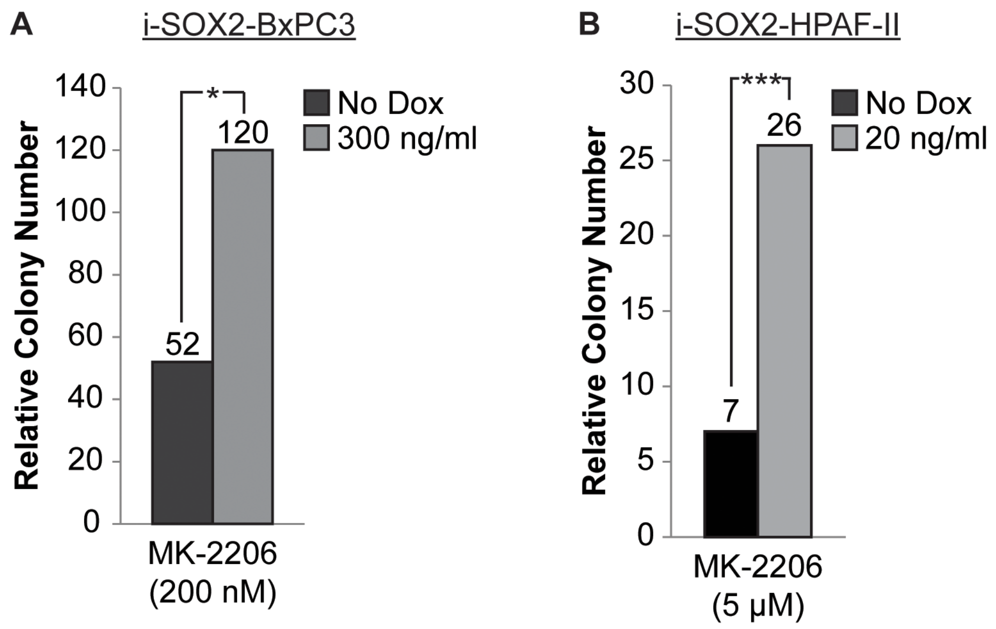

Supplementary Figure S7: Cloning efficiency of PDAC cells is reduced by treatment with MK-2206 and partially reversed by overexpression of SOX2. A. Clonal growth was determined by subculturing i-SOX2-BxPC3 **A.** and i-SOX2-HPAF-II **B.** cells at 80 cells per cm^2. After 8 days treatment with MK-2206 (200 nM and 5 µM, respectively) in the presence or absence of Dox colony numbers were determined. Statistical significance was determined by student's t-test (*$p<0.05$, ***$p<0.005$). The studies shown in A and B were repeated and similar results were obtained.

Supplementary Table S1 : Inhibitors used and the EC_{50} for each drug

Inhibitor Name	Target	Company, Location	T3M4	HPAF-II	BxPC3	L3.6
GSK-1120212, trametinib	MEK	Selleck, Houston, TX	40 nM	20 nM	4 nM	4 nM
AZD-6244, selumetinib	MEK	Selleck, Houston, TX	10 µM	ND	ND	ND
RDEA-119, refametinib	MEK	Active BioChem, Hong Kong	1 µM	ND	ND	ND
GDC-0623	MEK	Active BioChem, Hong Kong	20 nM	ND	ND	ND
GDC-0973, cobimetinib	MEK	Active BioChem, Hong Kong	200 nM	ND	ND	ND
MK-2206	AKT	Selleck, Houston, TX	2 µM	5 µM	200 nM	80 nM

*ND, not determined
